# In-situ synthesis of amorphous silver silicate/carbonate composites for selective visible-light photocatalytic decomposition

**DOI:** 10.1038/s41598-017-15405-6

**Published:** 2017-11-08

**Authors:** Ruya Cao, Hongcen Yang, Xiaolong Deng, Shouwei Zhang, Xijin Xu

**Affiliations:** School of Physics and Technology, University of Jinan, Shandong, 250022 P.R. China

## Abstract

Coupling two different semiconductors to form composite photocatalysts is an extremely significant technique for environmental remediation. Here, a one-step *in-situ* precipitation method has been developed to prepare amorphous silver silicate/carbonate (AgSiO/Ag_2_CO_3_) nanoparticles (NPs) composites, which are well dispersed sphere-like particles with the sizes of around ~50–100 nm. The high-efficiency photocatalytic activities under visible light (VL) have been carefully evaluated, and the AgSiO/Ag_2_CO_3_ NPs composites exhibit selective photocatalytic degradations on Methylene Blue (MB) and Rhodamine B (RhB). The maximum degradation rate for MB can reach ~99.1% within ~40 min under VL irradiation, much higher than that of RhB (~12%) in the same condition, which can be ascribed to (I) the smaller molecule size of MB than that of RhB, (II) the fast charge separation between AgSiO NPs and Ag_2_CO_3_ NPs, abundant heterojunction interfaces as well as fully exposed reactive sites. These composites are proposed to be an example for the preparation of other silicate composite photocatalysts for practical applications in environmental remediation.

## Introduction

Photocatalytic technology has become one of the most promising green technology as it can degrade organic pollutants in water to produce non-toxic substances such as CO_2_, H_2_O and so on with no secondary pollution, mild reaction conditions, and low energy consumption^1–3^. Especially, semiconductor photocatalysts have attracted wide attention in the field of environmental purification and a lot of achievements have been achieved^4,5^. Among them, silver carbonate (Ag_2_CO_3_) has received great research interests, but is restricted by its photocorrosion and lower photocatalytic degradation efficiency^2^. Therefore, it is significantly worthy to make great efforts to solve the problem for the real industrial application. Recently, it has been reported that introducing an electron acceptor in the photocatalytic reaction system can efficiently restrain the photocorrosion^3^. The silicates have been found to be the effective one^6,7^, which can not only enhance the photogenerated charge transfer but also broaden the spectral response range^6–8^. Furthermore, the excited electron–hole pairs can readily transfer between the particles due to the internal polar electric field in the silicate NPs. Among them, silver silicate (AgSiO) has been one of the most famous silicates due to its good stability, high efficiency, nontoxicity, low cost, and it has row band gap which can extend the large of light absorption^8^. Therefore, visible-light-driven photocatalysts can be exploited by coupling AgSiO with Ag_2_CO_3_ NPs. Furthermore, the heterojunctions by coupling them favor the separation of photogenerated electron-hole pairs resulting in the improvement of the photocatalytic activity^9,10^.

In this paper, AgSiO/Ag_2_CO_3_ composites with different ratios have been successfully synthesized by an *in-situ* method. The final samples with different molar ratios of SiO_3_^2−^ and CO_3_^2−^ exhibit the optimal photocatalytic activities. Besides, the selective photodegradation of two cationic dyes Methylene Blue (MB), Rhodamine B (RhB), and two another anionic dyes Methyl Orange (MO) and Congo Red (Cr) have been carefully investigated^11^. Finally, the photocatalytic mechanisms of these AgSiO/Ag_2_CO_3_ composites are carefully discussed.

## Experimental Sections

### Preparation of the amorphous silver silicates (AgSiO)

The AgSiO was synthesized via an *in-situ* method. For a typical synthesis, 0.2 M AgNO_3_ (40 mL) was added into 0.1 M Na_2_SiO_3_·9H_2_O (40 mL) under continuously stirring for 1.0 h. The precipitates were collected and washed with distilled water and ethanol, and finally dried at room temperature.

### Preparation of the silver carbonate (Ag_2_CO_3_)

0.2 M AgNO_3_ (40 mL) was slowly drop by drop added into 0.1 M NaHCO_3_ (20 mL) under continuously stirring for 1.0 h. The precipitates were collected and washed with distilled water and ethanol, and finally dried at room temperature.

### Preparation of the amorphous silver silicate/carbonate heterostructure (AgSiO/Ag_2_CO_3_)

The AgSiO/Ag_2_CO_3_ composites were synthesized via an *in-situ* precipitation method. In a typical synthetic procedure, 0.1 M Na_2_SiO_3_·9H_2_O (40 mL) were added to 0.1 M NaHCO_3_ (20 mL) under stirring for 5 min. Then, 0.2 M AgNO_3_ (40 mL) was slowly added to the mixture under stirring for 1.0 h. The precipitates were collected and washed with distilled water and ethanol, and dried at room temperature. The AgSiO/Ag_2_CO_3_ composites prepared with different molar ratios of Na_2_SiO_3_·9H_2_O and NaHCO_3_ in 40, 60, 80, 100 and 120 ml are denominated as AgSiO/Ag_2_CO_3_-2:1, AgSiO/Ag_2_CO_3_-3:1, AgSiO/Ag_2_CO_3_-4:1, AgSiO/Ag_2_CO_3_-5:1, and AgSiO/Ag_2_CO_3_-6:1, respectively.

### Characterization

Powder X-ray diffraction (XRD) patterns were collected using a D/MAX2500 V diffractometer equipped with Cu *K*_*α*_ radiation (λ = 1.5418 Å). The morphologies of the materials were observed using a FEI QUANTA FEG 250 field emission scanning electron microscope (SEM). The structural information of the samples was measured with the standard KBr disk method by a Fourier transform spectrophotometer (FT-IR, Avatar 370, thermo Nicolet). X-ray photoelectron spectroscopy (XPS) was carried out on ESCALAB250 with Mg *K*_*α*_ as the source and the C_1s_ peak at 284.6 eV as an internal standard. UV-vis diffuse reflection spectroscopy (DRS) was recorded with a Shimadzu UV-2500 spectrophotometer using BaSO_4_ as the reference. The Brunauer–Emmett–Teller (BET) specific surface areas were explored by nitrogen adsorption in a Micromeritics Tristar II 3020 nitrogen adsorption–desorption apparatus in accordance with the Barret–Joyner–Halenda (BJH) technique from the N_2_ adsorption isotherms.

### Photocatalytic tests

Photocatalytic degradation experiments were conducted in a photocatalytic reactor equipped with a 420 nm cut-off filter and a 500 W Xe lamp as the light source. For the photocatalytic reaction, the photocatalysts (20 mg) were mixed into a MB and RhB solution (50 mL, 10 mg/L). To guarantee the adsorption-desorption counterpoise, the mixed solution was stirred for 60 min in the darkness. Then, the solution was illuminated by VL and a small portion of the suspension (~3.0 mL) was sampled at 10 min illumination spacing. The catalytic efficiency was tested by using a UV-vis spectrophotometer (UV-2500, Shimadzu) for the degradation of RhB and MB.

## Results and Discussion

**Figure 1:**
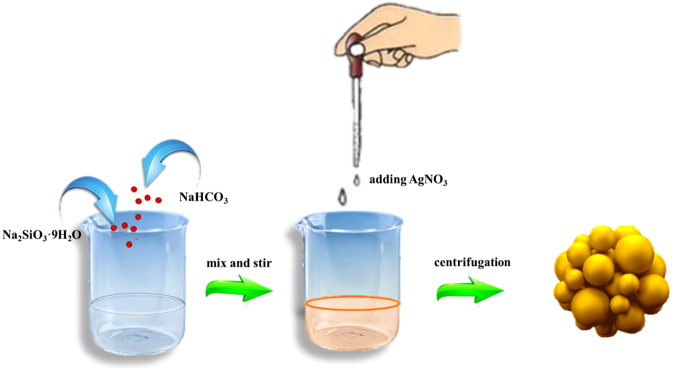
Schematic illustration of the synthesis of AgSiO/Ag_2_CO_3_ composites.

A facile *in-situ* precipitation method was designed to synthesize AgSiO/Ag_2_CO_3_ nanoparticles as illustrated in Fig. 1. The chemical reaction process can be described as follows:

1 $${{\rm{Ag}}}^{+}+{{\rm{SiO}}}_{3}^{2-}+{{\rm{CO}}}_{3}^{2-}+{{\rm{OH}}}^{-}\to {\rm{AgSiO}}/{{\rm{Ag}}}_{2}{{\rm{CO}}}_{3}+{{\rm{H}}}_{2}{\rm{O}}$$

By adding AgNO_3_ to the NaHCO_3_ solution, the CO_3_^2−^ present in the solution will react with the added Ag^+^ by electrostatic interaction to produce Ag_2_CO_3_. Then, the fixed Ag^+^ cations will further react with SiO_3_^2−^ to generate AgSiO with the addition of Na_2_SiO_3_ solution, resulting in the formation of AgSiO/Ag_2_CO_3_^5–7^.

**Figure 2:**
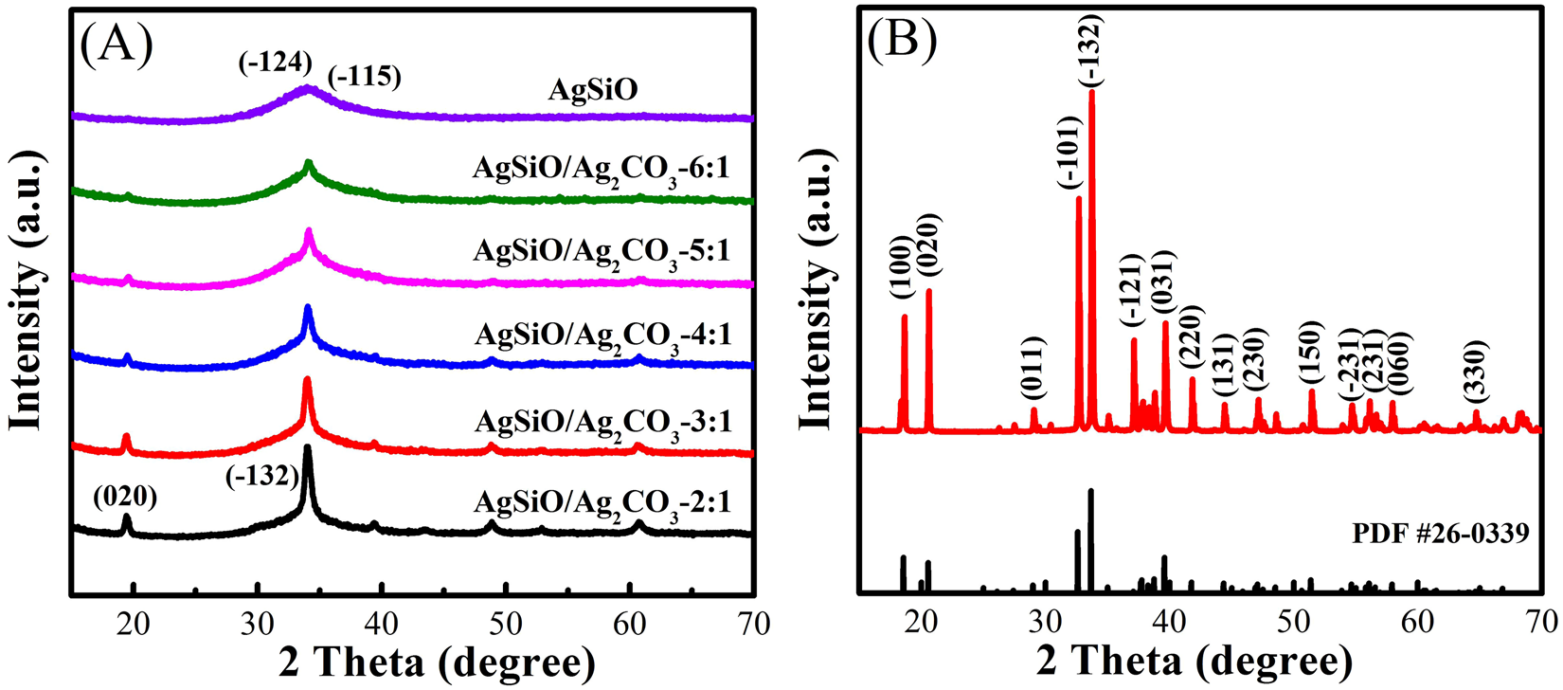
XRD patterns of (**A**) pure AgSiO and AgSiO/Ag_2_CO_3_ composites, (**B**) pure Ag_2_CO_3_.

The XRD patterns of pure AgSiO, Ag_2_CO_3_ and AgSiO/Ag_2_CO_3_ composites are shown in Fig. 2. The broad peak at about 34° could be indexed to the (−124) and (−115) planes for pure AgSiO in Fig. 2A, suggesting its amorphous structure^6^. For the pure Ag_2_CO_3_ NPs (Fig. 2B), the positions and intensities of the diffraction peaks are well matched with the standard JCPDS card^2,3,11^. The XRD pattern of AgSiO/Ag_2_CO_3_ composites is shown in Fig. 2A. Obviously, the diffraction peaks of AgSiO and Ag_2_CO_3_ NPs can be clearly found in curve and without any other peaks. The peaks of Ag_2_CO_3_ decreased with increasing AgSiO contents, and the characteristic peak intensity of AgSiO increases with the increase of the AgSiO contents, indicating that AgSiO/Ag_2_CO_3_ composites with high purity have been successfully synthesized by an *in-situ* precipitation^1,8^.

**Figure 3:**
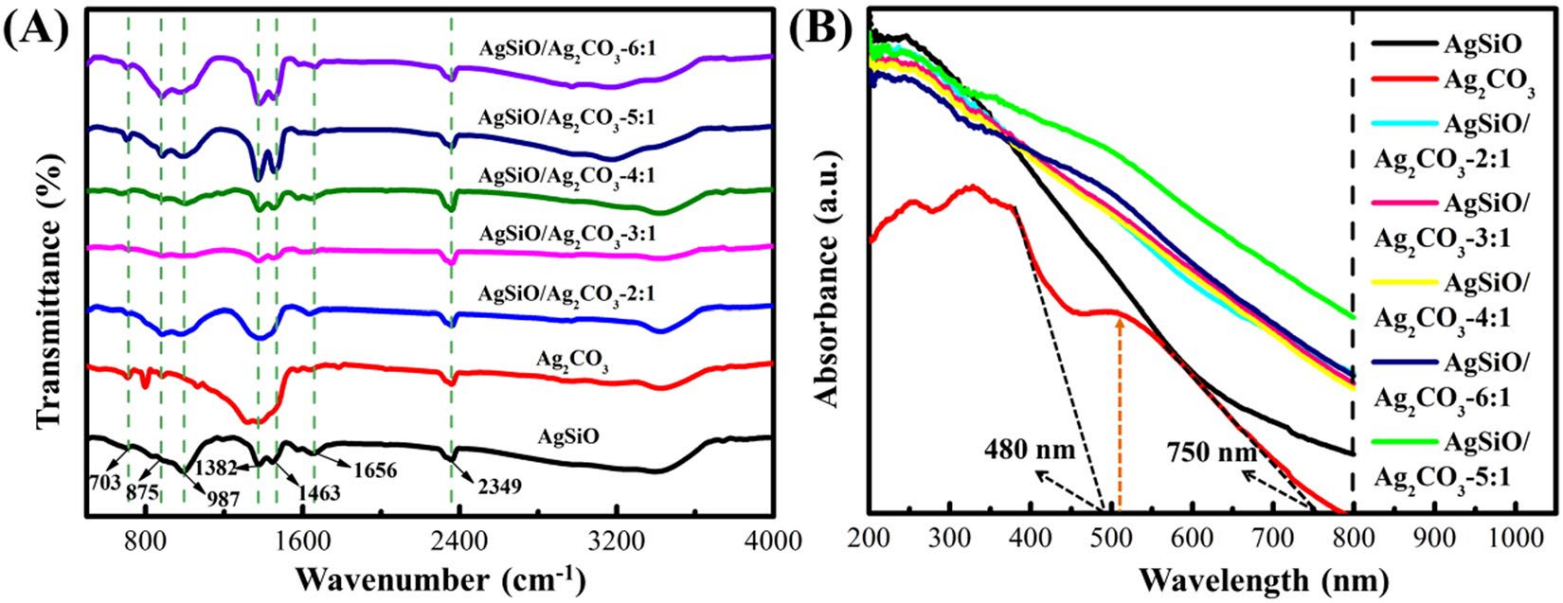
(**A**) FT-IR spectras of pure Ag_2_CO_3_, AgSiO and AgSiO/Ag_2_CO_3_ composites; (**B**) UV-vis diffuses reflectance spectra of Ag_2_CO_3_, AgSiO and AgSiO/Ag_2_CO_3_ composites.

FTIR spectra of AgSiO, Ag_2_CO_3_ and AgSiO/Ag_2_CO_3_ composites were recorded as shown in Fig. 3. In comparison to pure AgSiO, the absorption bands of AgSiO/Ag_2_CO_3_ composites obtained at around ~705 cm^−1^, ~893 cm^−1^, ~1382 cm^−1^, and ~1451 cm^−1^ are attributed to CO_3_^2−^ in Ag_2_CO_3_^1,10^. Compared with pure Ag_2_CO_3_, strong absorption band at around ~1382 cm^−1^ is found for AgSiO/Ag_2_CO_3_ composites, which belongs to the Si-O-Si stretching vibrations^8^. Moreover, there is also another shoulder peak indexed to the Si-O-Si bonds at ~1630 cm^−1^, which confirms the successful introduction of AgSiO in the composites. Nevertheless, the characteristic peak located at ~1382 cm^−1^ shifts to higher wavenumbers (Fig. 3). The observed blue shift in the composites after introducing AgSiO (Fig. 3) indicates the weakened bond strengths of Si-O-Si owing to the conjugation between Ag_2_CO_3_ and AgSiO^8,12^. This result demonstrates the strong interfacial coupling effect in the AgSiO/Ag_2_CO_3_ composites. Compared with the aggregated or large-size nanoparticles, the nature of AgSiO nanoparticles on Ag_2_CO_3_ acted as nano-islands can facilitate the formation of the heterojunction interfaces and guarantee the higher contact areas^13,14^. Both characters are essentials to promote photocatalytic activity and to enhance the separation efficiency of photogenerated charges.

The UV-vis diffuse reflectance spectra of AgSiO/Ag_2_CO_3_ composites together with pure AgSiO and Ag_2_CO_3_ are shown in Fig. 3. The light absorption edge of Ag_2_CO_3_ is measured to be ~480 nm, and mainly absorptions are ultraviolet light^14^. In addition, the peak at 520 nm can be indexed to the Ag nanocrystals. Therefore, we can see that a broad absorption ranging from ~480 to ~750 nm is detected, which is due to the generation of Ag nanocrystals^15,16^. The AgSiO/Ag_2_CO_3_ composites exhibit the stronger absorption than that of the Ag_2_CO_3_ NPs in both the visible and ultraviolet light region^17^. Obviously, the introduction of AgSiO can significantly enhance the absorption in the visible-light region and can even extend to near-infrared region, which is attributed to the SPR of Ag nanoparticles. It is inferred that the heterojunction of AgSiO/Ag_2_CO_3_ composites results in significantly decreased interfacial contact barrier and strengthened electronic coupling of the semiconductors to generate more photogenerated electrons/holes with improved photocatalytic performance^18,19^.

**Figure 4:**
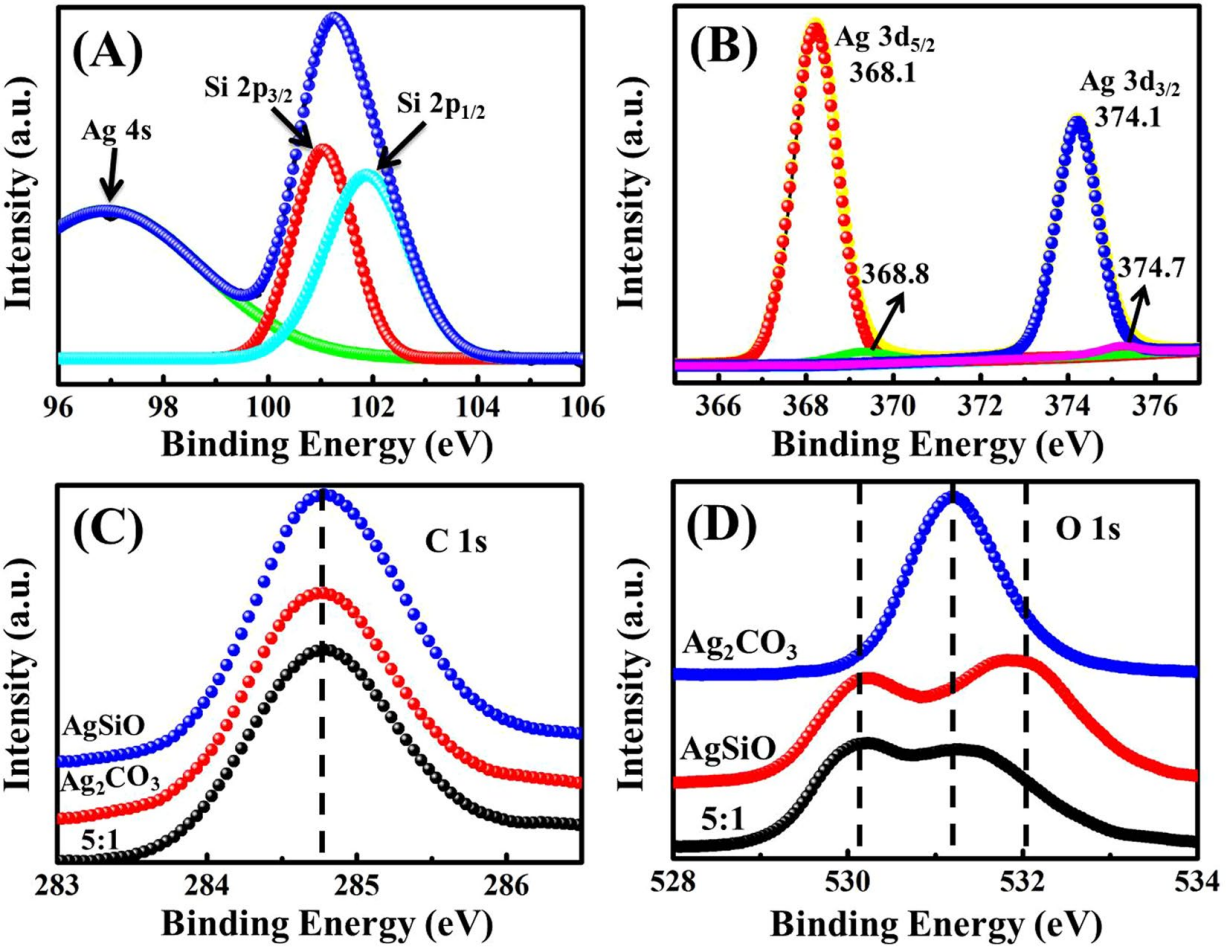
(**A**,**B**) main peaks of Si 2p_3/2_, Si 2p_1/2_ and Ag 3d for the AgSiO/Ag_2_CO_3_-5:1 composite; (**C**,**D**) high-resolution XPS spectra of C 1 s and O 1 s of the pure AgSiO, Ag_2_CO_3_ and AgSiO/Ag_2_CO_3_-5:1 composite.

The surface chemical compositions of AgSiO, Ag_2_CO_3_ and AgSiO/Ag_2_CO_3_-5:1 composite were investigated by XPS (Fig. 4). The full-scan XPS spectra of pure AgSiO, Ag_2_CO_3_ and AgSiO/Ag_2_CO_3_-5:1 composite (Figure S1) indicate the presence of Ag, Si, O in AgSiO, Ag, C, O in Ag_2_CO_3_ and Ag, Si, O, C in AgSiO/Ag_2_CO_3_-5:1 composite, respectively^15^. Figure 4A depictes the Si 2p peak of AgSiO/Ag_2_CO_3_-5:1 composite. The divided peaks located at ~96.9, ~102.8 and ~101 eV can be indexed into Ag 4 s, Si 2p_1/2_ and Si 2p_3/2_, respectively^6^. Figure 4B shows two XPS peaks located at ~368.1 eV and ~374.1, which can be indexed to Ag 3d_5/2_ and Ag 3d_3/2_ of pure AgSiO, Ag_2_CO_3_ and the AgSiO/Ag_2_CO_3_-5:1 composite^16,17^. These two peaks can be further divided into four peaks, ~368.1 and ~374.1 eV for Ag^+^ 3d_5/2_ and 3d_3/2_, and ~368.8 and ~374.7 eV for Ag^0^ 3d_5/2_ and 3d_3/2_, respectively. The peaks at ~368.8 and ~374.7 eV confirm the existence of metallic Ag^0^ in our AgSiO/Ag_2_CO_3_-5:1 composite. The carbon element in AgSiO is mostly ascribed to the adventitious hydrocarbon from XPS itself. Therefore the strength of C 1 s obeys the decreasing order of Ag_2_CO_3_ > AgSiO/Ag_2_CO_3_ > AgSiO^20^. Figure 4D demonstrates that O 1 s peak in AgSiO/Ag_2_CO_3_ contains two distinguishable shoulders in the spectrum, demonstrating that two chemical states of oxygen are present on the surface^21^. The O 1 s peak at ~530.18 eV can be ascribed to O in AgSiO. Another peak at ~531.93 eV is attributed to O in AgSiO and Ag_2_CO_3_^19^.

**Figure 5:**
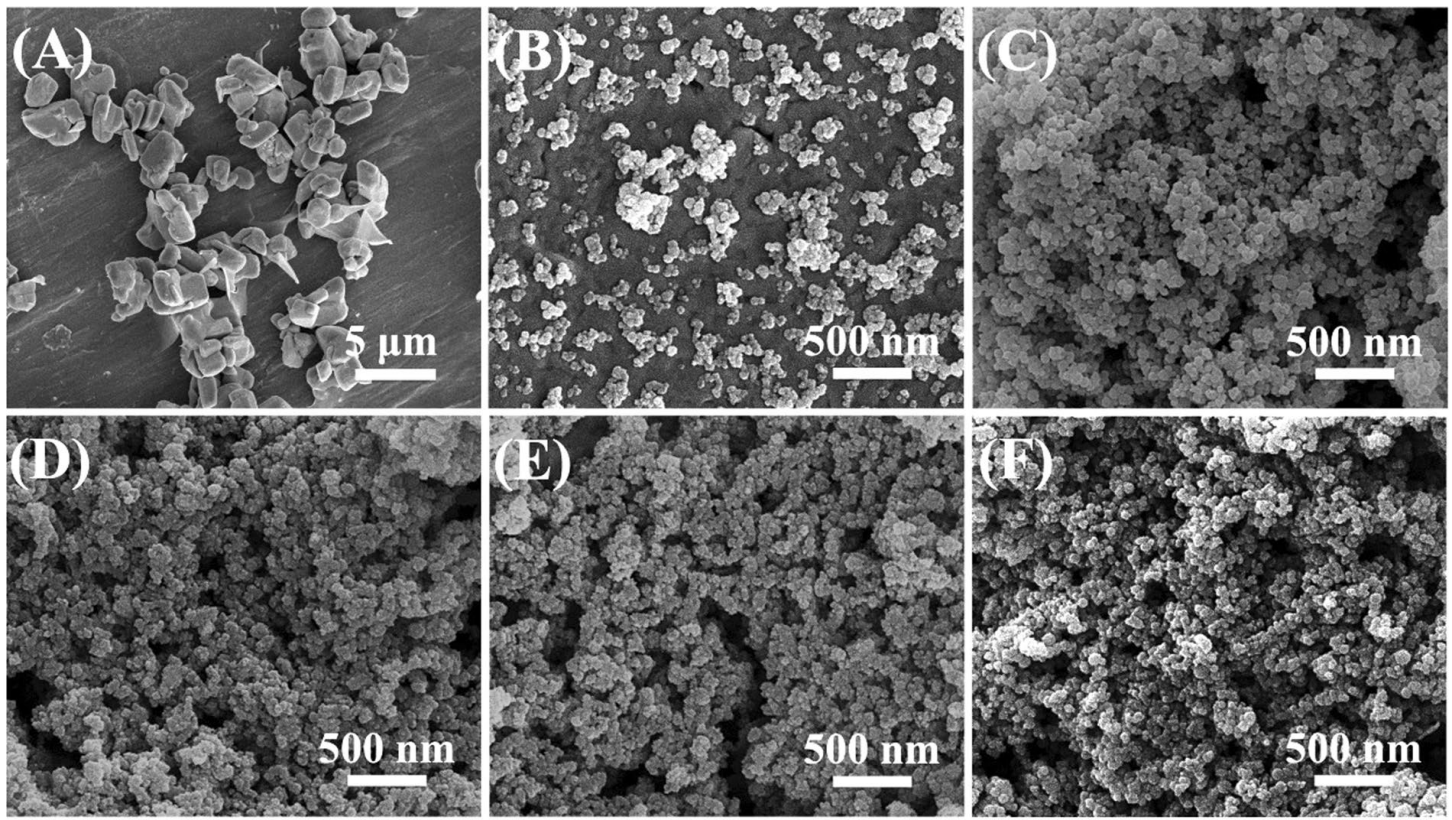
SEM images of (**A**) pure Ag_2_CO_3_, (**B**) pure AgSiO, (**C**) AgSiO/Ag_2_CO_3_-3:1 composite, (**D**) AgSiO/Ag_2_CO_3_-4:1 composite, (**E**) AgSiO/Ag_2_CO_3_-5:1 composite and (**F**) AgSiO/Ag_2_CO_3_-6:1 composite.

The morphologies of Ag_2_CO_3_, AgSiO and AgSiO/Ag_2_CO_3_ with different ratios are shown Fig. 5. It can be observed that Ag_2_CO_3_ are composed of microcubes with the length of ~1.0–5.0 μm in Fig. 5A ^22^. Figure 5B clearly shows that the AgSiO samples are sphere-like particles with the sizes of ~50–100 nm^22,23^. Figure 5C–F indicate that the morphologies of AgSiO/Ag_2_CO_3_ with different ratios are similar to AgSiO^24^. The presence of SiO^2−^ in the sources has great effects on the final morphology of composite. The particle sizes of the composite decrease with the increase addition of SiO^2−^. Furthermore, most particles are non-agglomerated and the sizes are mostly less than 100 nm, demonstrating that the samples are really nanosized cluster compounds^24^. The intimate contact between AgSiO and Ag_2_CO_3_ will strengthen the photogenerated charge separation and transfer^25^. Compared with traditional aggregated or large contact structures, this AgSiO/Ag_2_CO_3_ composites can not only provide more surface active sites for sequential photocatalytic reactions, but also shorten the migration distance of photogenerated charges^10^.

**Figure 6:**
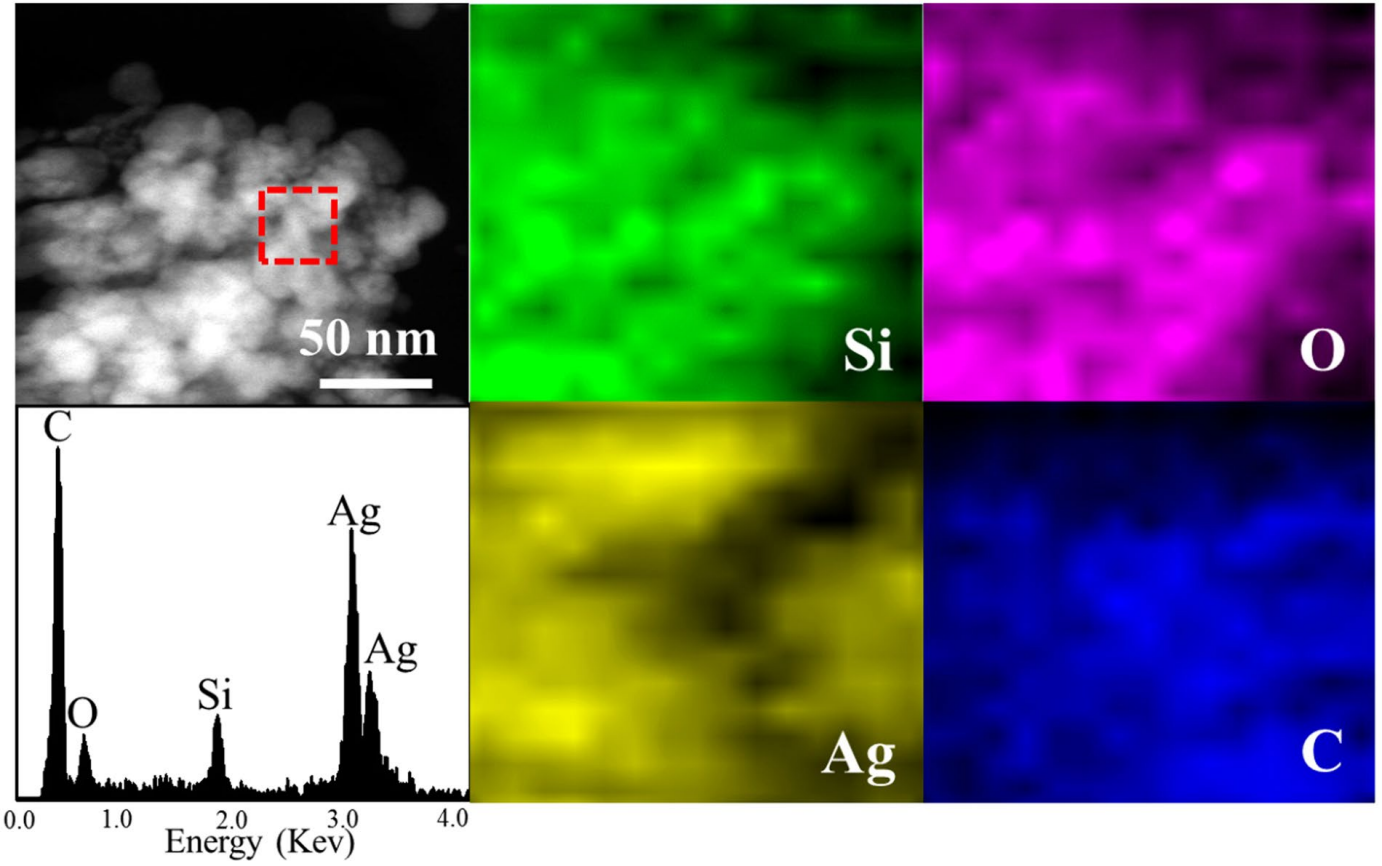
Elemental mapping images and EDS spectrum of the AgSiO/Ag_2_CO_3_-5:1 composite.

The HRTEM images in Figure S2(A,B) show that the size of AgSiO/Ag_2_CO_3_ NPs is of 10 to 20 nm. The lattices of AgSiO/Ag_2_CO_3_ NPs are clearly visible in the HRTEM images. The close interface between the AgSiO and Ag_2_CO_3_ nanoparticles reveals the formation of nano-heterojunction^26^. From the figure, we can see that Ag_2_CO_3_ is uniformly packaged by an amorphous AgSiO, limiting the growth of Ag_2_CO_3_^27^. Figure S2(B) shows the lattice spacing of uniformly dispersed Ag_2_CO_3_ nanoparticles. The lattice fringes of 0.275 nm is in agreement with the spacings of the (−101) plane of Ag_2_CO_3_ and consistent with JCPDS Card No. 26–0339^28^. No lattice fringes of AgSiO can be observed. This kind of heterojunction, which is favorable for the transport of photoexcited carriers, is formed between AgSiO and Ag_2_CO_3_^29^. Mainwhile, the composites can be further characterized by element mapping and EDS images (Fig. 6), where the Ag, Si, O and C elements are homogeneously distributed over the whole profile^30–32^. From the above results, we can expect that the strong interfacial coupling effect between AgSiO and Ag_2_CO_3_ will promote photogenerated electron-hole pairs separation and transfer, and thus further enhance the photocatalytic performance of AgSiO/Ag_2_CO_3_ composites^33^.

**Figure 7:**
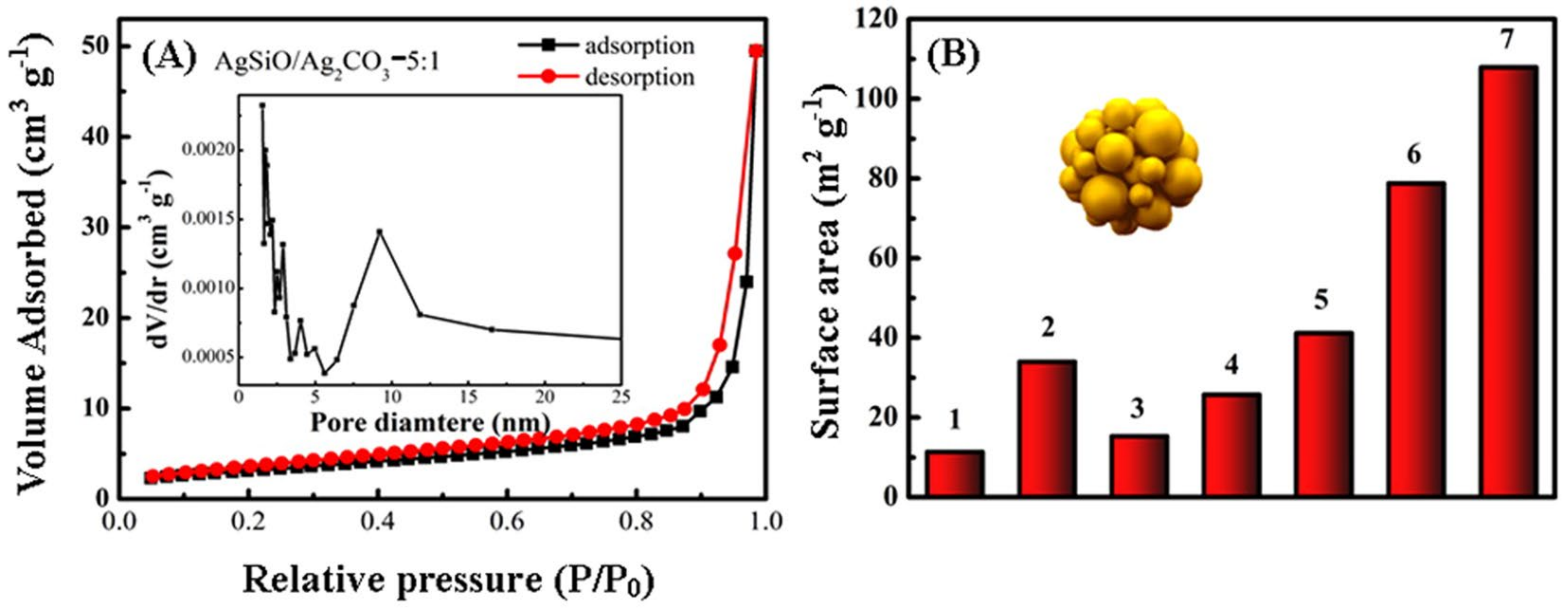
(**A**) N_2_ adsorption–desorption is otherms and pore size distribution curves calculated for AgSiO/Ag_2_CO_3_-5:1 composite; (**B**) surface area values of (1) Ag_2_CO_3_, (2) AgSiO, (3) AgSiO/Ag_2_CO_3_-2:1 composite,(4) AgSiO/Ag_2_CO_3_-3:1 composite, (5) AgSiO/Ag_2_CO_3_-4:1 composite, (6) AgSiO/Ag_2_CO_3_-5:1 composite and (7) AgSiO/Ag_2_CO_3_-6:1 composite.

Surface area, pore size and pore volume parameters for pure AgSiO, pure Ag_2_CO_3_ and AgSiO/Ag_2_CO_3_ composites were also investigated. The porosity of the AgSiO/Ag_2_CO_3_ composites sample is clearly enhanced. According to Figure S3, all the materials except for Ag_2_CO_3_ show a narrow pore size distribution with the average diameter of d > 2 nm^13,34^. With further increase of the AgSiO content, the adsorbed volume drops obviously indicating that the enhancement in adsorption volume is not driven solely by the small particles^6,13,35^. The surface areas of the different AgSiO/Ag_2_CO_3_ composites and pure AgSiO and Ag_2_CO_3_ are also calculated as shown in Fig. 7B for a better understanding of the composite nanostructure. It can be seen that the surface area of Ag_2_CO_3_ is ~11.4 m^2^·g^−1^. With further increasing of the amount of the AgSiO, the surface area values of the AgSiO/Ag_2_CO_3_ composites increase obviously. The surface area of AgSiO/Ag_2_CO_3_-6:1 composite can be achieved ~107.9 m^2^·g^−1^. Although the AgSiO/Ag_2_CO_3_-5:1 composite specific surface area isn’t the largest, the degradation is best, which can be attributed to its heterogeneous structure.

### Photocatalytic tests

**Figure 8:**
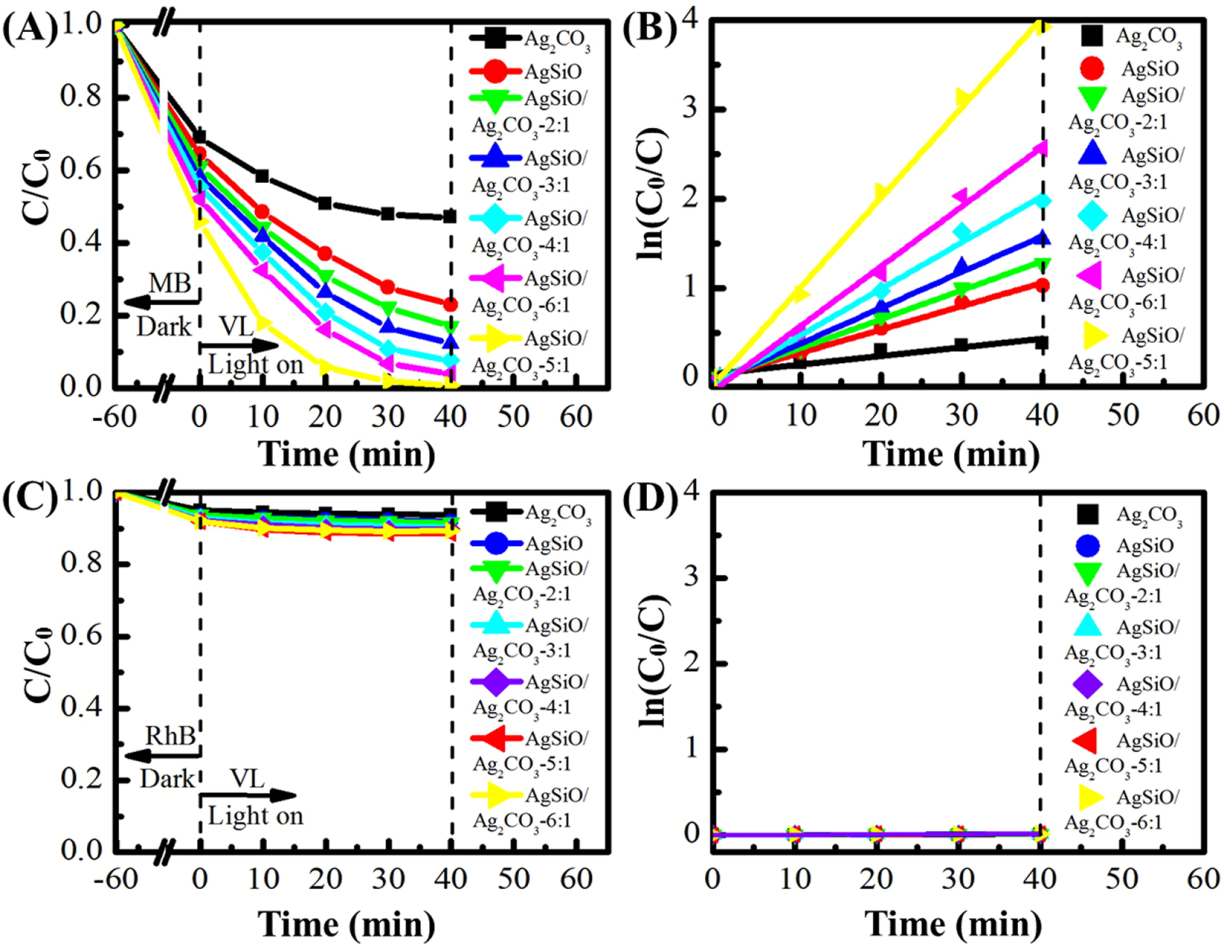
(**A**) The photocatalytic activities of the as-prepared photocatalysts for the degradation of MB under VL irradiation; (**B**) the rate constants of the as-prepared photocatalysts for the degradation of MB; (**C**) the photocatalytic activities of the as-prepared photocatalysts for the degradation of RhB under VL irradiation; (**D**) the rate constants of the as-prepared photocatalysts for the degradation of RhB.

The photocatalytic activities of pure AgSiO, Ag_2_CO_3_ and AgSiO/Ag_2_CO_3_ composites with different ratios under VL irradiation are shown in Fig. 8. The degradation reaches as ~99.1% under VL irradiation within ~40 min (Fig. 8A,B). The degradation of RhB is only ~12% in the same condition (Fig. 8C,D)^36,37^.

All AgSiO/Ag_2_CO_3_ composites exhibit higher photocatalytic activities than either AgSiO or Ag_2_CO_3_ with the order of AgSiO/Ag_2_CO_3_-5:1 > AgSiO/Ag_2_CO_3_-6:1 > AgSiO/Ag_2_CO_3_-4:1 > AgSiO/Ag_2_CO_3_-3:1 > AgSiO/Ag_2_CO_3_-2:1 > AgSiO > Ag_2_CO_3_, indicating the positive effect of AgSiO contents on enhancing the photocatalytic activities of the composites^8,37^. Full degradation of MB can be observed within ~40 min by VL irradiation in the presence of AgSiO/Ag_2_CO_3_-5:1 composite (Fig. 8A), illustrating the significantly improved photocatalytic activity of the AgSiO/Ag_2_CO_3_ composites^36^. However, further increment of proportion (AgSiO/Ag_2_CO_3_-6:1) results in decreased photocatalytic activity, which may be attributed to the recombination of photogenerated electrons and holes, and thenthe photocatalytic efficiency is restrained^38^.

**Figure 9:**
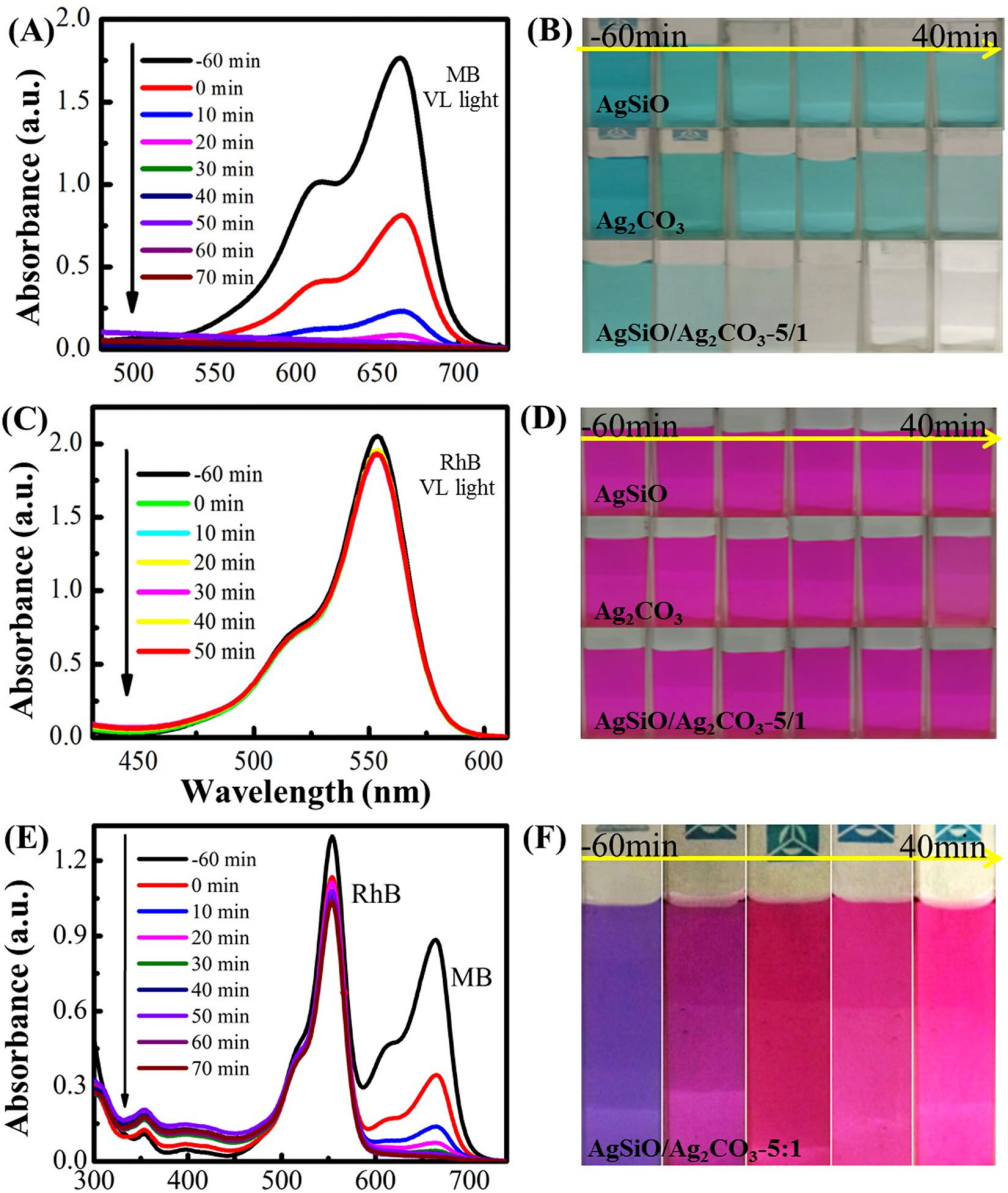
(**A**) The absorption spectra of MB degraded by AgSiO/Ag_2_CO_3_-5:1 composite under VL irradiation; (**B**) the corresponding digital photograph of MB degraded by AgSiO/Ag_2_CO_3_-5:1 composite under VL irradiation; (**C**) the absorption spectra of RhB degraded by AgSiO/Ag_2_CO_3_-5:1 composite under VL irradiation; (**D**) the corresponding digital photograph of RhB degraded by AgSiO/Ag_2_CO_3_-5:1 composite under VL irradiation; Absorption spectra (**E**) and corresponding digital photograph (**F**) of RhB and MB degraded by AgSiO/AgCO_3_-5:1 composite under VL irradiation.

Figure 9 shows the variation of the absorption spectra of MB under VL irradiation by AgSiO/Ag_2_CO_3_-5:1 composite. The characteristic peak intensities of MB gradually decrease by prolonging the irradiation time, and the adsorption peaks disappear within ~40 min irradiation^12,39^. The corresponding optical photographs of MB degradation using different photocatalysts under different irradiation times are collected and displayed in Fig. 9B. The color of MB gradually becomes lighter by prolonging the irradiation time, it can be seen that the color of AgSiO/Ag_2_CO_3_-5:1 composite becomes transparent while pure color for AgSiO and Ag_2_CO_3_ turns into azury^40^. The variation of the absorption spectra of RhB under VL irradiation by AgSiO/Ag_2_CO_3_-5:1 composite is shown in Fig. 9C. The characteristic peak intensities of RhB gradually decreased by prolonging the irradiation time, and the adsorption peaks became constant within ~40 min irradiation indicating the very slow degradation of RhB^12,38^. These corresponding optical photographs of RhB degradation using different photocatalysts under different irradiation times are in Fig. 9D, showing that the color of RhB almost keeps unchanged.

The selective photocatalytic phenomenon of the AgSiO/Ag_2_CO_3_-5:1 composite was examined by photodegradation of MB and RhB^38^. The absorptions peaks of MB located at ~664 nm almost disappear after irradiation within ~40 min. However, the absorption peaks of RhB located at ~554 nm doesn’t change too much (Fig. 9)^39^. As for two cationic dyes MB and RhB, we speculate that the discrepancy in adsorption capacities among them can be ascribed to the molecule size^29,35^. That is, because the size of MB molecules is smaller than those of RhB, MB can be intercalated into the space, while RhB are too large to intercalate into the NPs^37^.

Similarly, we used MB&MO, MO&RhB and MO&CR mixture dyes to further research the selectivity of the material. As shown from the figure S4, When AgSiO/Ag_2_CO_3_ composites were added into the binary mixtures, respectively, the characteristic peaks of cationic dyes MB disappeared quickly, while the characteristic peaks of all anionic dyes remained unchanged^41^. These results demonstrated excellent selective removal of cationic dyes over anionic dyes due to the electrostatic effect^11,12^.

### Photocatalytic mechanism

**Figure 10:**
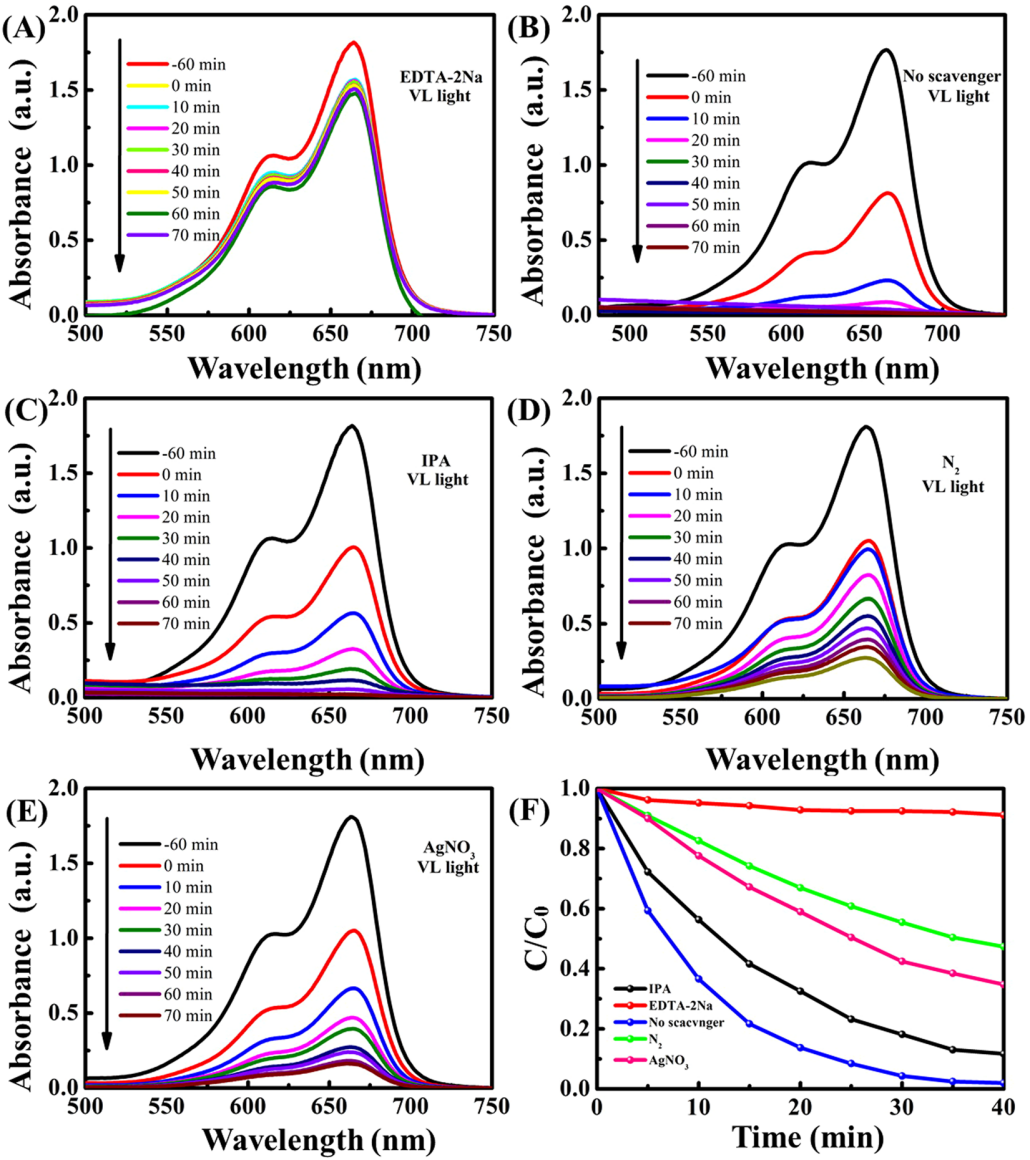
The typical visible absorption spectra (**A**–**E**) and concentration changes (**F**) of MB over AgSiO/Ag_2_CO_3_-5:1 composite in the presence of EDTA-2Na, IPA, N_2_ and in the absence scavengers.

The electrons and holes produced by photocatalysis have strong reduction and oxidation capacities. The main active species of different photocatalysts may vary due to their different band structure and phase compositions^10,38^. Thus, to explore the mechanism of the high photocatalytic activities and to assess the contribution of the reactive species, trapping experiments of reactive species were conducted using ethylenediaminetetraacetate (EDTA-2Na), iso-propyle alcohol (IPA) and N_2_ as h^+^ and OH^−^ and e^−^ scavengers, respectively^10,16^. By adding scavengers into the degradation solutions, the reactive species in the degradation process can be revealed. As shown in Fig. 10, the degradation rate decreases clearly to ~8.8% in the presence of EDTA-2Na (h^+^ scavenger) and the degradation rate is ~98% in the absence of scavengers, which indicates that h^+^ is the major reactive specie for MB degradation (Fig. 10A,B)^8,40^. Introducing IPA displays a significant effect on the K_app_. It decrease from ~0.102 min^−1^ to ~0.055 min^−1^ (Fig. 10C), suggesting that the radical is also a dominant reactive species. And the degradation rate decreases obviously to ~53% and ~66% in the presence of N_2_ (O^2−^ scavenger) and AgNO_3_ (e^−^ scavenger), which suggests that O^2−^ and e^−^ is the partly reactive species for MB degradation (Fig. 10D,E). Through Fig. 10F, we can visually see that the O^2−^, e^−^ and h^+^ are reactive species^42^.

**Figure 11:**
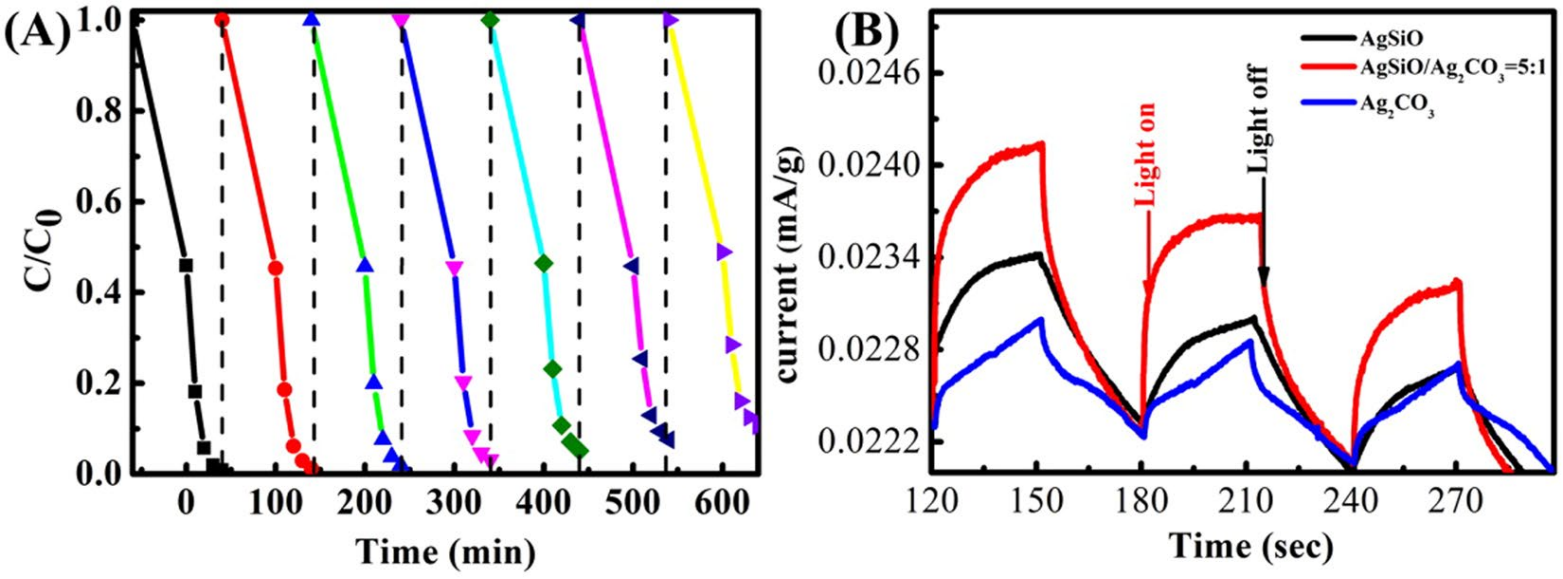
(**A**) The photocatalytic stability of AgSiO/Ag_2_CO_3_-5:1 composite in recycling reactions; Photocurrent response curves of pure Ag_2_CO_3_, AgSiO and AgSiO/Ag_2_CO_3_ composites under visible light irradiation (**B**).

The recyclability of the photocatalysts is significant factors in their practical applications. Figure 11A indicates that the recyclability for AgSiO/Ag_2_CO_3_-5:1 composite. After six successive cycles, AgSiO/Ag_2_CO_3_-5:1 composite still possesses ~89% degradation rate of MB after ~40 min VL irradiation, indicating its high recyclability^30,39,43^. The results indicate that the incorporation of AgSiO with Ag_2_CO_3_ photocatalyst successfully improves the VL photocatalytic performance and restrains the photocorrosion in a large level^13,44^. To further comprehend the separation and recombination of electron-hole pairs in pure AgSiO, Ag_2_CO_3_ and AgSiO/Ag_2_CO_3_ composites, the photocurrent test is carried out under visible light^33^. In this study, electrochemical and photoelectrochemical measurements were performed in 1 M Na_2_SO_4_ electrolyte solution in a three-electrode quartz cell. Pt sheet was used as a counter electrode and Hg/Hg_2_Cl_2_/sat. KCl was used as a reference electrode. The pure AgSiO, Ag_2_CO_3_ and AgSiO/Ag_2_CO_3_ composites on ITO was used as the working electrode for investigation. The photoelectrochemical response was recorded with a CHI 660E electrochemical system^33^. The photocurrent-potential plots of these samples are shown in Fig. 11B. The figure shows the obvious photocurrent intensity of different samples under illumination by 250 lumens LED for 30 second intervals. The best photocurrent intensity (0.7 μA·g^−1^) of AgSiO was obtained when the applied potential is 1.3 V. And the best photocurrent intensity of Ag_2_CO_3_ was 1.2 μA·g^−1^. However, the photocurrent intensity was improved to 1.8 μA·cm^−1^ with the same applied potential after a heterojunction formed form AgSiO and Ag_2_CO_3_, which was three times greater than pure Ag_2_CO_3_. This phenomenon reveals that the AgSiO/Ag_2_CO_3_ heterostructures possess a larger carrier concentration than the pure AgSiO and Ag_2_CO_3_ NPs, and more electron-hole pairs are generated for the charge separation process^45^. These results of the photocurrent tests are in agreement with the results of the photodegradation of MB. The obvious photocurrent demonstrates that the interfacial charge separation between AgSiO and Ag_2_CO_3_ NPs exists in this composite^46^.

**Figure 12:**
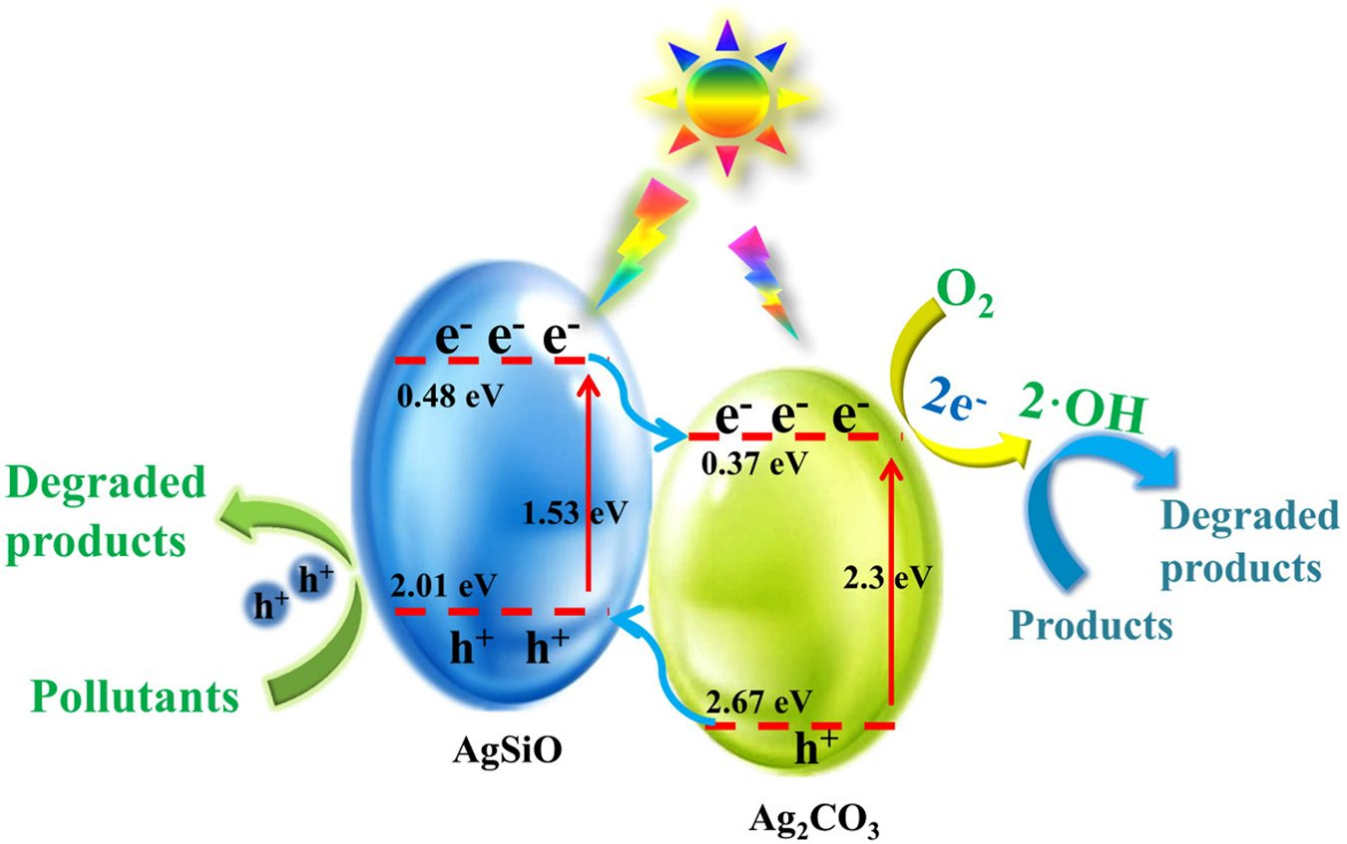
Proposed mechanisms of photogenerated charge transfer and pollutants degradation in the AgSiO/Ag_2_CO_3_-5:1 composite under VL irradiation.

Based on experimental results, Fig. 12 depicts a diagrammatic sketch for photocatalytic mechanism. Under VL irradiation, AgSiO can absorb VL, leading to the excitation of e^−^ to the conduction band (CB) and whilst keeping h^+^ in the valence bands (VB)^2,7^. For AgSiO/Ag_2_CO_3_ heterojunctions, the photogenerated e^−^ on the CB of AgSiO can easily migrate to the CB of Ag_2_CO_3_ while the photogenerated h^+^ in the VB of Ag_2_CO_3_ migrates to AgSiO^19^. That is to say, the appropriately aligned band edges of AgSiO and Ag_2_CO_3_ indicates that the migration of effective photogenerated charges can occur via the heterojunctions with strong interfacial coupling effect in the composite^47^. The migration of photogenerated charges limit the transmission of photogenerated e^−^ and h^+^ on different sides, which reduces the recombination rate of photogenerated electron-hole pairs and improves the abundance and stability of photogenerated charge in the composite^6,7^. At the same time, the isolated photogenerated charges promote the production of reactive oxidative species, i.e. •O^2−^ and •OH, which are responsible for degrading MB confirmed by Fig. 12 ^26,48,49^.

## Conclusions

In summary, a facile *in-situ* precipitation method has been designed and developed to synthesize a series of AgSiO/Ag_2_CO_3_ composites with the sizes in the range of 100 nm. The as-synthesized AgSiO/Ag_2_CO_3_-5:1 composite shows superior VL photocatalytic activities, and the degradation of MB reach as ~99.1% under VL irradiation within ~40 min, which can be ascribed to the synergetic effect between AgSiO and Ag_2_CO_3_, including the maximum heterojunction interface with intimate contact, enhanced photogenerated charge separation efficiency, fully exposed reactive sites as well as excellent VL response in the composite. For the selectivity for degradation, we speculate that the discrepancy in degradation capacities among the two anionic dyes can be ascribed to the molecule size. This work will give insights into the importance of rational design of heterojunction systems, and provide a potential method for the construction of efficient heterojunction photocatalysts with controllable sizes and space distributions.

## Electronic supplementary material

Supplementary Information
