# Supplementary material for: In-situ synthesis of amorphous silver silicate/carbonate composites for selective visible-light photocatalytic decomposition
Source: Sci Rep. 2017 Nov 8;7:15001. doi: 10.1038/s41598-017-15405-6 (PMC5678140; doi:10.1038/s41598-017-15405-6)
Supplement: Supplementary file 1 — Supplementary Information [file 41598_2017_15405_MOESM1_ESM.pdf]

In-situ synthesis of amorphous silver silicate/carbonate composites for selective visible-light photocatalytic decomposition

Ruya Cao, Hongcen Yang, Xiaolong Deng, Shouwei Zhang*, Xijin Xu*

School of Physics and Technology, University of Jinan, Shandong 250022, PR China

E-mail: sps_xuxj@ujn.edu.cn; zhangsw-1122@163.com

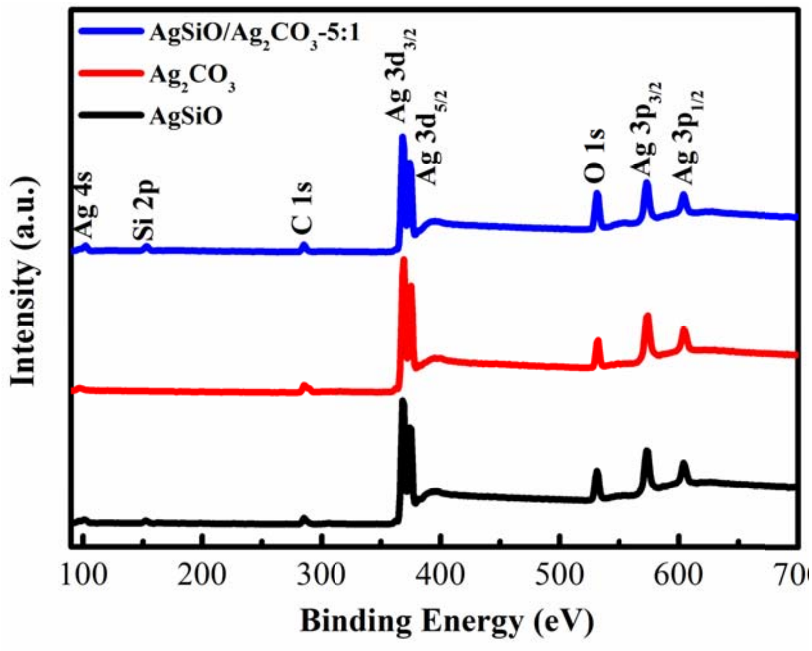

Figure S1. The complete XPS spectra of pure AgSiO, Ag_2CO_3 and AgSiO/Ag_2CO_3-5:1 composite.

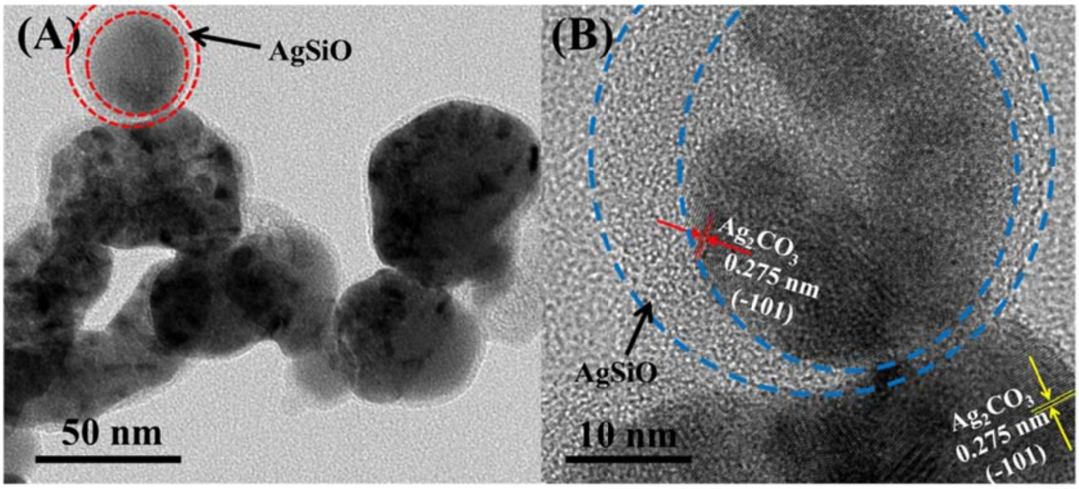

Figure S2. (A-B) HRTEM images and lattice fringes of AgSiO/Ag_2CO_3 composites.

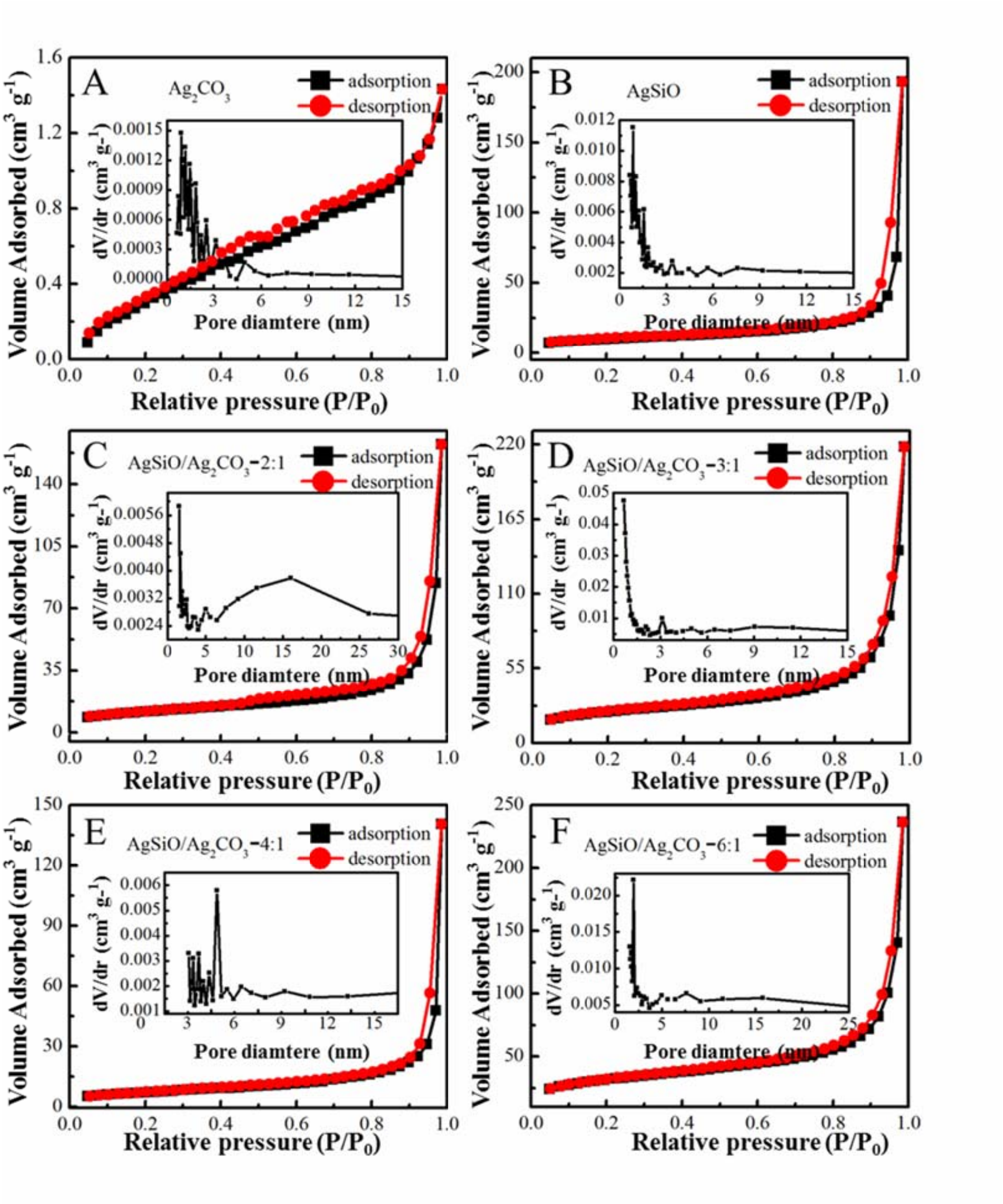

Figure S3. N_2 adsorption–desorption isotherms and pore size distribution curves calculated for (A) Ag_2CO_3, (B) AgSiO, (C-F) $AgSiO/Ag_2CO_3$ composites.

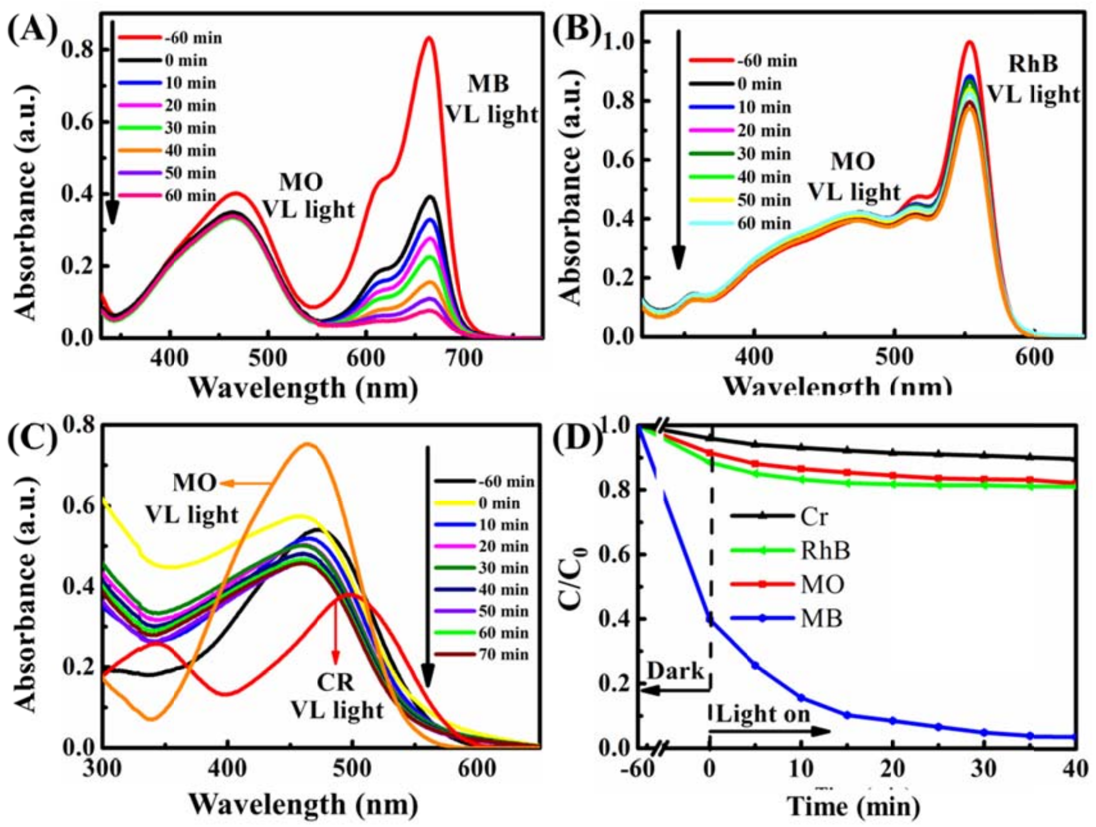

Figure S4. The absorption spectra changes of AgSiO/Ag_2CO_3-5:1 composite under VL irradiation: (A) MB& MO; (B) MO& RhB; (C) MO& CR. (D) The photocatalytic activities of the degradation of MB, RhB, MO and CR.
